# 2504. Online Clinician Education Results in Performance Changes in Clinical Practice Related to Hepatitis B Vaccination

**DOI:** 10.1093/ofid/ofad500.2122

**Published:** 2023-11-27

**Authors:** Arun S Nair, Amy Larkin, Nimish Mehta

**Affiliations:** Medscape Education, TARRYTOWN, New York; Medscape Education, TARRYTOWN, New York; Medscape Education, TARRYTOWN, New York

## Abstract

**Background:**

This study examined whether online continuing medical education (CME/CE) focused on evidence-based best practices for implementing hepatitis B vaccination guidelines into clinical care would result in the adoption of new clinical practices.

**Methods:**

Clinicians participated in an online, 30-minute CME/CE video discussion among experts with synchronized slides. Performance in the real world was assessed by inviting learners to complete a survey 30-60 days post-education, identifying practice changes and the degree to which clinicians experience barriers to those changes. For each possible practice, learners reported whether they were implementing for the first time or had modified it due to education, they were already doing it prior to education; or they were not doing it before or after education. They also indicated barriers they experience at least “some” of the time for each practice. The data were collected from 5/19/2022 to 10/24/2022.

**Results:**

There were 44 learners consisting of PCPs, Diab/Endos, Ob/Gyns, Nurses, NPs and Pharmacists who completed the survey. Analysis showed that 100% of learners made a practice change or had practices reinforced due to education. Top 5 practice changes modified or implemented as a result of education included:

- Utilize key differences between available hepatitis B vaccines to counsel and appropriately vaccinate patients (67%)
- Apply the Advisory Committee on Immunization Practices (ACIP) recommendations for hepatitis B vaccination (66%)
- Implement the latest universal vaccination recommendations for the hepatitis B vaccine (59%)
- Identifying patients who are candidates for hepatitis B vaccination (59%)

Some of the main barriers to implementing practice changes identified by the learners include:

- Patient misinformation, inaccurate expectations, and vaccine hesitancy
- Multiple doses, decreased adherence, and patients being lost to follow up
- Understanding the indications for various hepatitis B vaccines
- Time restraints of staff and training needs

**Conclusion:**

The practice changes identified in this assessment provide a compelling evidence that participation in online CME/CE prompts adoption of changes in practice related to hepatitis B vaccination. Future education is needed to address the barriers identified in this assessment.

**Disclosures:**

**All Authors**: No reported disclosures

